# Differentiation of Promonocytic U937 Cells to Monocytes Is Associated with Reduced Mitochondrial Transport of Ascorbic Acid

**DOI:** 10.1155/2018/4194502

**Published:** 2018-02-08

**Authors:** Maddalena Scotti, Mara Fiorani, Andrea Guidarelli, Orazio Cantoni

**Affiliations:** Department of Biomolecular Sciences University of Urbino “Carlo Bo”, 61029 Urbino, Italy

## Abstract

Growth of promonocytic U937 cells in the presence of DMSO promotes their differentiation to monocytes. After 4 days of culture in differentiating medium, these cells ceased to proliferate, displayed downregulated ryanodine receptor expression, and responded to specific stimuli with enhanced NADPH-oxidase-derived superoxide formation or cytosolic phospholipase A_2_-dependent arachidonic acid release. We found that the 4-day differentiation process is also associated with downregulated SVCT2 mRNA expression, in the absence of apparent changes in SVCT2 protein expression and transport rate of ascorbic acid (AA). Interestingly, under the same conditions, these cells accumulated lower amounts of the vitamin in their mitochondria, with an ensuing reduced response to external stimuli sensitive to the mitochondrial fraction of AA. Further analyses demonstrated an unexpected increase in mitochondrial SVCT2 protein expression, however, associated with reduced SVCT2-dependent AA uptake in isolated mitochondria. A decrease in the transporter Vmax, with no change in affinity, was found to account for this response. Differentiation of promonocytic cells to monocytes is therefore characterized by decreased SVCT2 mRNA expression that, even prior to the onset of SVCT2 protein downregulation or apparent changes in plasma membrane transport activity, impacts on the mitochondrial accumulation of the vitamin through a decreased Vmax of the transporter.

## 1. Introduction

Ascorbic acid (AA), the reduced form of vitamin C, is transported in most cell types through high-affinity/low-capacity Na^+^-dependent transporter 1 (SVCT1) and 2 (SVCT2) [1–3]. Under these conditions, cells accumulate high concentrations of the vitamin that can be further transported within specific organelles in which these transporters are also expressed [4]. In this direction, we recently provided evidence for the expression of functional SVCT2 in U937 cell mitochondria [5, 6]. This transporter, unlike its plasma membrane counterpart [1–3], was surprisingly characterized by a high affinity, since virtually Ca^2+^-independent and maximally stimulated by low millimolar concentrations of Na^+^ [6]. An additional important observation was that the activity of both the plasma membrane and mitochondrial SVCT2 is susceptible to inhibition by low micromolar levels of dehydroascorbic acid (DHA) [7, 8], the oxidized form of vitamin C. DHA levels in biological fluids are generally very low, as a consequence of its poor stability and, most importantly, because of its rapid uptake mediated by facilitative hexose transporters [9]. It can therefore be suggested that the DHA-dependent inhibition of plasma membrane and mitochondrial SVCT2 activities may eventually take place under conditions associated with superoxide formation, with a net inhibition of vitamin C transport at low DHA levels, and with the possibility of a switch in the uptake mechanisms, when the availability of DHA is significantly enhanced [10, 11].

These findings document a specific strategy employed by U937 cells to transport AA through the plasma and mitochondrial membranes, possibly susceptible to modification by events associated with their differentiation to monocytes. Numerous studies have indeed addressed a similar question in various cell types, however exclusively focusing on the cellular expression of SVCT2 and on the cellular uptake of the reduced form of the vitamin. Enhanced SVCT2 expression was observed during the process of myoblast differentiation to myotubes [12, 13] as well as in differentiating osteoblasts [14–17] and neurons [18, 19]. Other studies have shown that the process of PMA-induced differentiation of THP-1 cells to macrophages is accompanied by enhanced SVCT2 mRNA/protein expression and AA transport activity [20]. While the importance of AA transport in macrophages has been emphasized by additional observations [21], much less is known on monocytes, except that these short-lived circulating cells normally accumulate very large amounts of vitamin C. The reported concentrations are in the 2–6 mM range [22, 23], that is, about two order of magnitude greater than those found in erythrocytes [24].

The present study was performed with the aim of investigating the previously unexplored issue of the impact of the differentiation of promonocytic cells to monocytes on the expression and activity of the plasma membrane and mitochondrial SVCT2.

## 2. Materials and Methods

### 2.1. Chemicals

Arachidonyl trifluoromethyl ketone (AACOCF3) was from Calbiochem (San Diego, CA, USA). AA, dithiothreitol (DTT), tetrabutylammonium hydrogen sulfate (TBA), ethylenediaminetetraacetic acid (EDTA), cytochalasin B (cyt B), choline chloride, 4-hydroxymercuribenzoic acid (pCMB), sulfinpyrazone (S-pyr), rotenone, myxothiazol, caffeine (Cf), A23187, dimethyl sulfoxide (DMSO), diphenyleneiodonium (DPI), apocynin, phorbol-12-myristate-13-acetate (PMA), DL-buthionine-[S,R]-sulfoximine (BSO), ryanodine (Ry), and the remaining chemicals were from Sigma-Aldrich (Milan, Italy). [^3^H] Arachidonic acid was from Amersham Pharmacia Biotech (Buckinghamshire, England). MitoSOX red and Rhod 2-acetoxymethyl (AM) were purchased from Molecular Probes (Leiden, The Netherlands). Perkin-Elmer Life and Analytical Sciences (Boston, MA) supplied L-[1-^14^C]AA (specific activity 5.35 mCi/mmol), which was dissolved in deionized water containing 0.1 mM acetic acid and stored in multiple aliquots at −20°C until use [20].

### 2.2. Cell Cultures, Treatment Conditions, and Assessment of Cellular AA Uptake

U937 cells were cultured in suspension in RPMI 1640 medium (Sigma-Aldrich, Milan, Italy) supplemented with 10% foetal bovine serum (Euroclone, Celbio Biotecnologie, Milan, Italy), penicillin (100 units/ml), and streptomycin (100 *μ*g/ml) (Euroclone), at 37°C in T-75 tissue culture flasks (Corning, Corning, NY) gassed with an atmosphere of 95% air-5% CO_2_. These cells were differentiated to monocytes by a 4-day growth in culture medium supplemented with 1.3% DMSO, as previously described [25].

Prior to experiments, the undifferentiated and differentiated cells were counted and resuspended in extracellular buffer (EB, 15 mM Hepes, 135 mM NaCl, 5 mM KCl, 1.8 mM CaCl_2_, 0.8 mM MgCl_2_, and pH 7.4) at a density of 1 × 10^6^ cells/ml. AA was dissolved in the same buffer, supplemented with appropriate amounts of [^14^C] AA and immediately utilized in uptake experiments performed as detailed in [6].

In some experiments, the cells were first preloaded with AA and then exposed to either BSO or peroxynitrite. The BSO experiments were performed using 35 mm dishes containing 5 × 10^5^ cells resuspended in complete RPMI medium. The peroxynitrite experiments were instead performed using 15 ml plastic tubes containing 5 × 10^5^ cells in prewarmed saline A (140 mM NaCl, 5 mM KCl, 4 mM NaHCO_3_, and 5 mM glucose; pH 7.4), as previously described [26].

Myxothiazol was dissolved in 95% (*v*/*v*) ethanol. Rotenone was dissolved in DMSO (0.05%).

### 2.3. Isolation of Mitochondria and Assessment of Mitochondrial AA

Mitochondria were isolated and resuspended in intracellular buffer (IB, 15 mM HEPES-sodium, 15 mM NaCl, 120 mM KCl, 1 mM MgCl_2_, and pH 7.6). Uptake studies involved a 3 min exposure to [^14^C] AA and were terminated by sudden addition of 1 ml of ice-cold IB, containing an excess of unlabeled AA [27]. Transport kinetic parameters were calculated using the Michaelis-Menten equation and the linear transformation of Eadie-Hofstee. Details are provided in [6].

In some experiments, the cells were first exposed to AA and then processed to isolate the mitochondria to determine the fraction of the vitamin associated with these organelles. Details of this procedure and on HPLC method employed for the assessment of AA content are reported elsewhere [6].

### 2.4. Subcellular Fractionation and Western Blot Analysis

The cells were lysed immediately after the treatments [28] or processed to obtain the mitochondrial fraction as indicated above. Details on the Western blotting apparatus and conditions are reported elsewhere [28]. The antibodies against SVCT1 (N-20, sc-9924), SVCT2 (S-19, sc-9926), actin (C-2, sc-8432), and HSP-60 (H-1, sc-13115), as well as the horseradish peroxidase-conjugated secondary antibody, were purchased from Santa Cruz Biotechnology (Santa Cruz, CA). Antibodies against actin and HSP-60 were used to assess the equal loading of the lanes and the purity of the fractions.

### 2.5. Measurement of ROS

The cells were supplemented with either 10 *μ*M DHR or 5 *μ*M MitoSOX red (30 min) prior to the end of the treatments. The cells were then processed as detailed in [29] and analyzed with a fluorescence microscope (BX-51, Olympus, Milan, Italy), equipped with a SPOT-RT camera unit (Diagnostic Instruments, Delta Sistemi, Rome, Italy).

### 2.6. Measurement of Mitochondrial Ca^2+^

Cells were preexposed (30 min) to Rhod 2-acetoxymethyl ester (10 *μ*M), treated, and then analyzed with a fluorescence microscope as detailed elsewhere [30].

### 2.7. Release of [^3^H] Arachidonic Acid

The cells were grown for 18 h in a medium containing [^3^H] arachidonic acid (0.5 *μ*Ci/ml), washed with saline A, and finally resupplemented at a density of (2 × 10^5^ cells/ml) in 1 ml saline A supplemented with 1 mg/ml fatty acid-free bovine serum albumin. After treatments, the cell suspension was centrifuged and the radioactivity was determined in the supernatant as previously described [25].

### 2.8. Cytotoxicity Assay

Cytotoxicity was determined with the trypan blue exclusion assay [31].

### 2.9. Measurement of DNA Single-Strand Breakage by the Alkaline Halo Assay

DNA single-strand breakage was determined using the alkaline halo assay developed in our laboratory [32].

### 2.10. Reverse Transcriptase-Polymerase Chain Reaction (RT-PCR)

Total RNA was extracted with Trizol reagent (Invitrogen) according to the manufacturer's instructions and quantified with NanoDrop (Thermo Scientific, DE, USA); 1 *μ*g of total RNA was pretreated with Dnase I (Sigma-Aldrich) and used for cDNA synthesis with the SMARTScribe Reverse Transcriptase (Clontech Laboratories, Mountain View, CA, USA). The following primers were used to analyze the expression of SVCT1: 5′-GCCCCTGAACACCTCTCATA-3′ and Rev 5′-ATGGCCAGCATGATAGGAAA-3′; SVCT2: 5′-TTCTGTGTGGGAATCACTAC-3′ and Rev 5′-ACCAGAGAGGCCAATTAGGG-3′. Amplification of GAPDH was used for internal loading control. The PCR reaction mixture was prepared with 100 nM of forward and reverse primers, 2X PCR Master Mix Kit (DIATHEVA, Fano, Italy) and 50 ng of cDNA for each sample. The PCR conditions were one cycle at 95°C for 8 min, 35/40 cycles at 95°C for 15 s, 57°C for 45 s, and 72°C for 45 s and one final cycle at 72°C for 7 min. Amplification products were examined by electrophoresis on 1.5–2% agarose gels and visualized with ethidium bromide.

### 2.11. GSH Assay

Cellular nonprotein thiols were assayed as described in [33], with minor modifications. Since GSH represents more than 90% of the nonprotein thiols, the latter will be referred to as GSH. In short, the pellet obtained after washing the cells (4 × 10^6^) three times with phosphate-buffered saline (136 mM NaCl, 10 mM Na_2_HPO_4_, 1.5 mM KH_2_PO_4_, and 3 mM KCl; pH 7.4) was resuspended in 150 *μ*l of a solution containing 1.67% (*v*/*v*) metaphosphoric acid [0.2% EDTA and 30% (w/v) NaCl] and kept for 5 min at ice-bath temperature. The samples were then centrifuged for 5 min at 10,000 ×g, and the GSH content was determined in the supernatant at 412 nm by a spectrophotometric assay, using 5,5′-dithiobis(2-nitrobenzoic acid)(ε412 = 13,600 M^−1^ cm^−1^).

### 2.12. Statistical Analysis

The results are expressed as means ± SD. Statistical differences were analyzed by one-way ANOVA followed by Dunnett's test for multiple comparisons or two-way ANOVA followed by Bonferroni's test for multiple comparison. A value of *p* < 0.05 was considered significant.

## 3. Results

### 3.1. Accumulation of Ascorbic Acid in Undifferentiated and Differentiated U937 Cells

**Figure 1:**
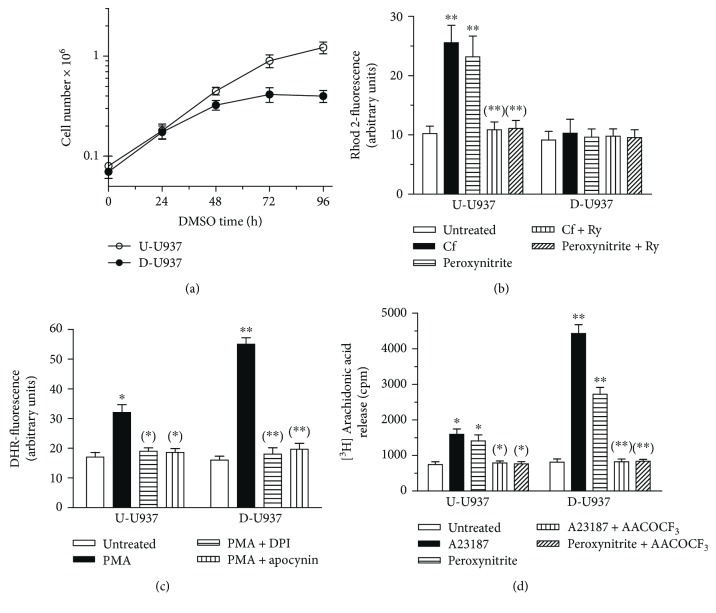
Characterization of the U937-derived differentiated cells. (a) Counts of U937 cells exposed for increasing time intervals to 0 (open circles) or 1.3% DMSO (closed circles). (b) Undifferentiated (U-U937) and differentiated (D-U937) cells were preloaded with Rhod 2-AM, treated for 5 min with 0 or 20 *μ*M Ry, and then exposed for 10 min to either 10 mM Cf or 200 *μ*M peroxynitrite. Rhod 2-fluorescence was then quantified as detailed in Materials and Methods. Results represent the means ± SD calculated from at least 3 separate experiments. ^∗∗^*p* < 0.001 as compared to untreated cells, ^(∗∗)^*p* < 0.001 as compared to cells treated with Cf or peroxynitrite (one-way ANOVA followed by Dunnett's test). (c) U-U937 and D-U937 cells were exposed for 15 min to 1 *μ*M DPI or 10 *μ*M apocynin and subsequently treated for 30 min with 100 *μ*g/ml PMA. After treatments, the cells were analyzed for DHR-fluorescence. Results represent the means ± SD calculated from at least 3 separate experiments. ^∗^*p* < 0.01 and ^∗∗^*p* < 0.001 as compared to untreated cells, ^(∗)^*p* < 0.01 and ^(∗∗)^*p* < 0.001 as compared to cells treated with PMA (one-way ANOVA followed by Dunnett's test). (d) [^3^H] arachidonic acid-labeled U-U937 and D-U937 cells were exposed for 5 min with 0 or 50 *μ*M AACOCF_3_ and then treated for 10 min with 10 *μ*M A23187 or 200 *μ*M peroxynitrite. After the treatments, [^3^H] arachidonic acid release was quantified as described in Materials and Methods. Results represent the means ± SD calculated from at least 3 separate experiments. ^∗^*p* < 0.01 and ^∗∗^*p* < 0.001 as compared to untreated cells, ^(∗)^*p* < 0.01 and ^(∗∗)^*p* < 0.001 as compared to cells treated with AA23187 or peroxynitrite (one-way ANOVA followed by Dunnett's test).

Promonocytic U937 cells are conveniently differentiated to monocytes when grown in the presence of DMSO [25]. Under these conditions, cells cease to proliferate between day 2 and 3 (Figure 1(a)), become smaller, and experience important biological changes, as the loss of expression of the Ry receptor, which can be detected at day 4 [34]. An immediate consequence of this modification is the inability of these cells, from now on defined as differentiated cells, to respond to 10 mM Cf or 200 *μ*M peroxynitrite, with an increased Ry-sensitive mitochondrial accumulation of Ca^2+^, instead detected in undifferentiated cells (Figure 1(b)). An additional, important characteristic acquired by the differentiated U937 cells is an increased NADPH oxidase activity, measured in terms of PMA-dependent DHR-fluorescence response, sensitive to two different NADPH oxidase inhibitors, DPI, and apocynin (Figure 1(c)). DHR is a general fluorescence probe-detecting O_2_^−^ as well as H_2_O_2_ [35]. Finally, we also obtained evidence of increased phospholipase A_2_ activity [25], readily detected in terms of arachidonic acid release after stimulation with 10 *μ*M A23187 or 200 *μ*M peroxynitrite (Figure 1(d)). AACOCF_3_, a well-established inhibitor of cytosolic phospholipase A_2_ [36], suppressed arachidonic acid release detected under both circumstances.

**Figure 2:**
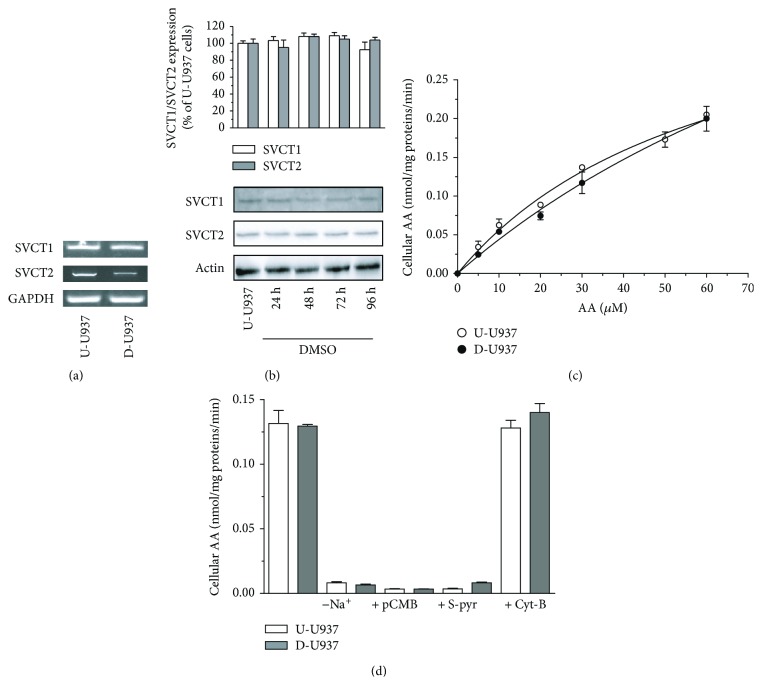
SVCT1 and SVCT2 expression and cellular uptake of AA in undifferentiated and differentiated cells. (a) RT-PCR analysis of SVCT1 and SVCT2 mRNA in undifferentiated (U-U937) and differentiated (D-U937) cells. (b) SVCT1 and SVCT2 protein expression determined by Western blot analysis of samples obtained from U937 cells grown for 0–96 h in the presence of DMSO. Anti-actin antibodies were used to provide an internal loading control. Relative amounts of SVCT1 and SVCT2 were determined by densitometric analysis of three different experiments and are expressed as % of U-U937 cells. (c) Vitamin C content in U-U937 and D-U937 cells exposed for 5 min to 0–60 *μ*M AA. (d) Effect of Na^+^ omission (and replacement with choline), pCMB, S-pyr, and cyt B on AA transport in U-U937 and D-U937 cells exposed for 5 min to 30 *μ*M AA. Results represent the means ± SD calculated from at least three separate experiments.

After this initial characterization, we investigated the impact of the differentiation process on SVCT1 and SVCT2 mRNA expression. The results illustrated in Figure 2(a) provide evidence for a significant downregulation of the transporter characterized by a greater affinity, SVCT2, with hardly any effect detected in the case of SVCT1. It was also interesting to observe that the reduced SVCT2 mRNA expression did not bear detectable consequence in terms of SVCT2 protein expression during the 4 days of growth in differentiating medium (Figure 2(b)). A similar observation was made by measuring SVCT1 protein expression. Next, we performed uptake experiments in which the cells were exposed for 5 min to increasing concentrations of AA in a DTT-containing buffer. Under these conditions, similar rates of AA uptake were observed in undifferentiated and differentiated cells (Figure 2(c)). In addition, AA transport was in both cell types entirely dependent on Na^+^-AA cotransporter(s), was indeed insensitive to cyt B (25 *μ*M), an inhibitor of glucose/DHA transporters [37], and suppressed by Na^+^ omission, as well as by pCMB (40 *μ*M) or S-pyr (200 *μ*M) (Figure 2(d)).

The above results are therefore indicative of a similar uptake of the reduced form of the vitamin in undifferentiated and differentiated U937 cells, despite the observed downregulation of SVCT2 mRNA expression.

### 3.2. Lower Rates of AA Accumulation in the Mitochondria of Differentiated U937 Cells

**Figure 3:**
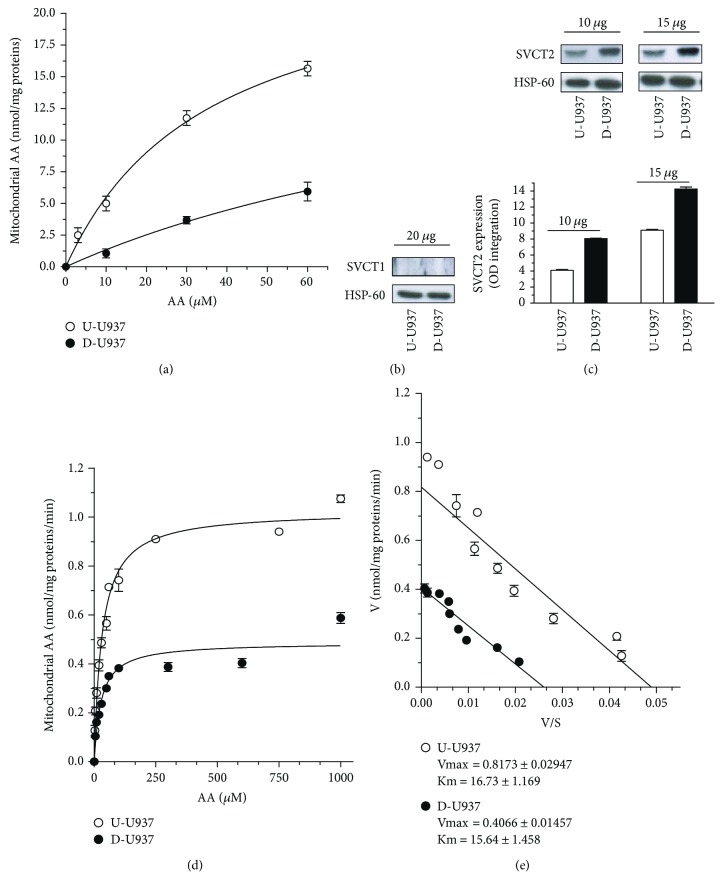
Mitochondrial uptake of AA in undifferentiated and differentiated cells. (a) Vitamin C content of mitochondria isolated from undifferentiated (U-U937) and differentiated (D-U937) cells immediately after exposure (10 min) to 0–60 *μ*M AA. (b) Western blot analysis of U-U937 and D-U937 cell-derived mitochondrial lysates (20 *μ*g of mitochondrial proteins) using anti-SVCT1 antibodies. (c) Western blot analysis of U-U937 and D-U937 cell-derived mitochondrial lysates (10 and 15 *μ*g of mitochondrial proteins) using anti-SVCT2 antibodies. Anti-HSP-60 antibodies were used to provide an internal loading control and for comparative densitometric analysis. Results represent the means ± SD calculated from at least three separate experiments. (d) Vitamin C content in mitochondria isolated from U-U937 and D-U937 cells and then exposed for 3 min to 0–1000 *μ*M [^14^C]-AA. Results represent the means ± SD calculated from at least three separate experiments. (e) Eadie-Hofstee plot of the data in C with the calculated Vmax and Km values.

We next tested the impact of the differentiation process on the mitochondrial transport of the vitamin. For this purpose, the differentiated and undifferentiated cells were exposed to increasing concentrations of AA and then processed for the isolation of mitochondria that were finally analyzed for their vitamin C content. The results illustrated in Figure 3(a) indicate that the fraction of mitochondrial AA is remarkably lower in the differentiated cells than in their undifferentiated counterpart.

These results indicate that the process of U937 cell differentiation is paralleled by a decreased ability to take up AA in mitochondria.

### 3.3. Increased SVCT2 Protein Expression in the Mitochondria of Differentiated U937 Cells

We tested whether the decreased mitochondrial accumulation of AA observed in differentiated cells was due to reduced expression of mitochondrial SVCT2. As the first step, we reproduced our previous findings [5, 6] documenting the lack of expression of SVCT1 in the mitochondria of undifferentiated cells and demonstrated that the same is true for their differentiated counterpart (Figure 3(b)). We then moved back to the use of anti-SVCT2 antibodies and employed two different amounts of mitochondrial proteins, as indicated in Figure 3(c). Under both conditions, we obtained the unexpected and apparently contradictory result of enhanced SVCT2 expression in the mitochondria of the differentiated cells. Although not reported in the present study, the purity of the mitochondrial preparations is routinely determined, as described in our recent paper [6]. We confirmed the absence of cross-contamination between mitochondrial and plasma membranes, in which SVCT2 is also expressed. There was instead some cross-contamination with the endoplasmic reticulum, in which however we had no evidence of SVCT2 expression. Identical results were obtained in undifferentiated and differentiated cells (not shown).

### 3.4. Differentiation of U937 Cells Is Associated with Decreased Vmax of Mitochondrial SVCT2

In order to investigate the reasons of the observed dichotomy between reduced mitochondrial uptake of AA and greater mitochondrial SVCT2 immunoreactivity detected in differentiated cells, we decided to perform AA uptake studies in isolated mitochondria. Preliminary experiments revealed that the rate of AA uptake is linear in the first 5 min of exposure in the mitochondria derived from both the undifferentiated and differentiated cells (not shown). Under these conditions, the results obtained in concentration-response studies were best described by two hyperbolic curves, saturating at >60 *μ*M AA, however with a remarkably lower accumulation of the vitamin observed in differentiated cell mitochondria (Figure 3(d)). Analysis of the transport data by the Eadie-Hofstee method produced straight lines (Figure 3(e)), consistently with the presence of a single functional component in the mitochondria of each cell type. Although apparent Km values were similar (16.73 ± 1.169 *μ*M and 15.64 ± 1.458 *μ*M for undifferentiated and differentiated U937 cell mitochondria, resp.), Vmax values were in fact significantly lower for the differentiated (0.406 ± 0.015 nmol/mg proteins/min) versus undifferentiated (0.817 ± 0.029 nmol/mg proteins/min) cells.

### 3.5. The Effects of AA Supplementation in Undifferentiated and Differentiated U937 Cells Exposed to BSO or Peroxynitrite

The well-established notion that AA is both an antioxidant and a scavenger of various reactive species [23, 38] implies that conditions associated with a significant mitochondrial accumulation of the vitamin effectively counteract the deleterious effects mediated by agents eliciting mitochondrial superoxide formation. Our results previously obtained in U937 cells exposed to arsenite [29, 31], under conditions exclusively associated with mitochondrial superoxide formation [29, 39], are in keeping with this notion. Indeed, a short-term (10 min) preexposure to as low as 10 *μ*M AA abolished mitochondrial superoxide formation and the downstream deleterious effects leading to MPT-dependent apoptosis [29, 31]. We therefore addressed the question of whether the differentiated cells require incubation with greater concentrations of AA to acquire a resistant phenotype, but immediately realized that this approach was complicated by the intrinsic resistance of these cells to the metalloid. Arsenite, even at a 4-fold greater concentration, failed to promote mitochondrial superoxide formation and toxicity in differentiated cells (not shown). While the reasons of this resistance are currently under investigation, we preferred to avoid the use of greater concentrations of the metalloid, since the interpretation of the experimental results would have been complicated by the recruitment of different mechanisms, for example, related to the binding of arsenite to protein thiols.

**Figure 4:**
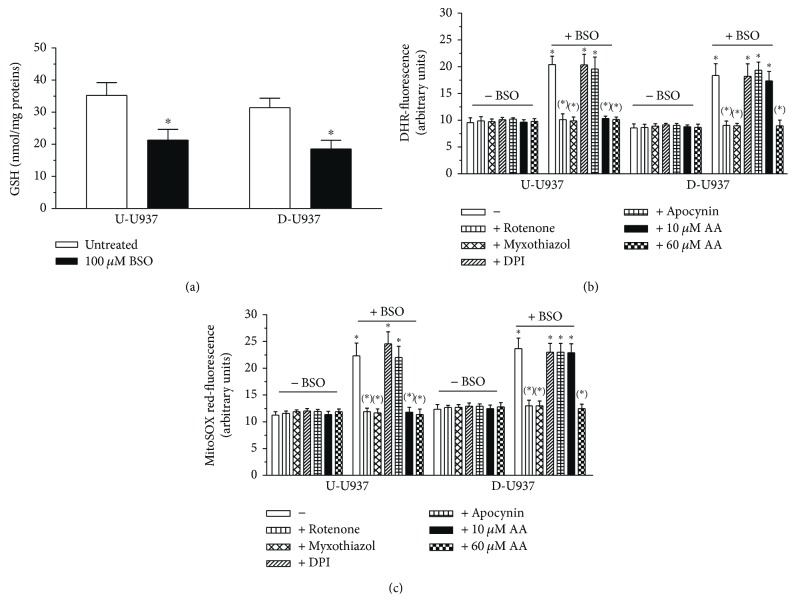
Mitochondrial superoxide formation induced by BSO: different concentrations of AA are required to promote similar protective effects in undifferentiated and differentiated cells. (a) Undifferentiated (U-U937) and differentiated (D-U937) cells were exposed for 4 h to 0 or 100 *μ*M BSO. After treatments, the cells were analyzed for their GSH content. (b and c) The cells were treated for 4 h with BSO, in the absence or presence of rotenone (0.5 *μ*M), myxothiazol (5 *μ*M), DPI (1 *μ*M), and apocynin (10 *μ*M). These inhibitors were given to the cells 3 min prior to addition of BSO. Experiments were also performed in cells preexposed for 10 min to 10 *μ*M or 60 *μ*M AA prior to the 4 h exposure to BSO. The effect of AA or other inhibitors in the absence of BSO was also tested. After treatments, the cells were analyzed for DHR-fluorescence (b) or MitoSOX red-fluorescence (c). ^∗^*p* < 0.001 as compared to untreated cells and ^(∗)^*p* < 0.001 as compared to cells preexposed to AA and treated with BSO (one-way ANOVA followed by Dunnett's test).

We therefore decided to move to a different paradigm based on the use of a high concentration of BSO (100 *μ*M), an inhibitor of *γ*-glutamylcysteine synthetase [40]. As indicated in Figure 4, a 4 h treatment of undifferentiated cells with BSO partially reduced cellular GSH (A) and caused significant DHR (B) and MitoSOX red (C) fluorescence responses. Note that, while DHR is responsive to various reactive species generated both in the intra- and extramitochondrial compartments, MitoSOX red only detects superoxide formation in the mitochondria of live cells [41]. The results illustrated in Figure 4 indicated that the responses detected with either DHR (B) or MitoSOX red (C) were prevented by rotenone, a complex I inhibitor [42], as well as by myxothiazol, an inhibitor of the electron flow from the reduced coenzyme Q to cytochrome *c_1_* [43], and were instead insensitive to apocynin or DPI.

These results therefore suggest that BSO selectively promotes mitochondrial superoxide formation, with hardly any contribution mediated by the NADPH oxidase. Apocynin and DPI indeed failed to affect the formation of reactive species generated by BSO (Figures 4(b) and 4(c)) and suppressed superoxide formation elicited by PMA-dependent stimulation of NADPH oxidase activity (Figure 1(c)). Interestingly, as indicated in Figures 4(b) and 4(c), the effects of rotenone or myxothiazol were mimicked by a low concentration of AA (10 *μ*M), supplemented under conditions resulting in significant mitochondrial vitamin C accumulation (Figure 3(a)).

The results obtained with the differentiated cells were identical in terms of BSO-dependent loss in cellular GSH (Figure 4(a)) and formation of reactive oxygen species (Figures 4(b) and 4(c) which, based on inhibitor studies, also appeared to be represented by mitochondrial superoxide. The differentiated cells, however, unlike the undifferentiated cells, were not sensitive to treatment with 10 *μ*M AA. A 6-fold greater concentration of the vitamin was indeed necessary to suppress BSO-dependent superoxide formation in these cells. Figures 4(b) and 4(c) also provide results obtained with the different agents used to modulate the effect of BSO under conditions in which the inhibitor of GSH synthesis was omitted. None of these treatments, including rotenone or myxothiazol, produced detectable effects under these conditions.

We then moved to another approach to document the consequences of the reduced mitochondrial accumulation of AA in the differentiated cells. It is very well established that physiological concentrations of the vitamin, besides being involved in cytoprotective mechanisms, can also be engaged in specific reactions leading to enhanced responses to specific reactive species [11, 44–46]. As an example, we found that preexposure of U937 cells to AA enhances their susceptibility to the deleterious effects mediated by various hydroperoxides, in particular peroxynitrite [11, 46]. We also found that the mitochondrial fraction of AA is specifically linked to the enhanced cyto-genotoxicity induced by peroxynitrite [30, 46].

**Figure 5:**
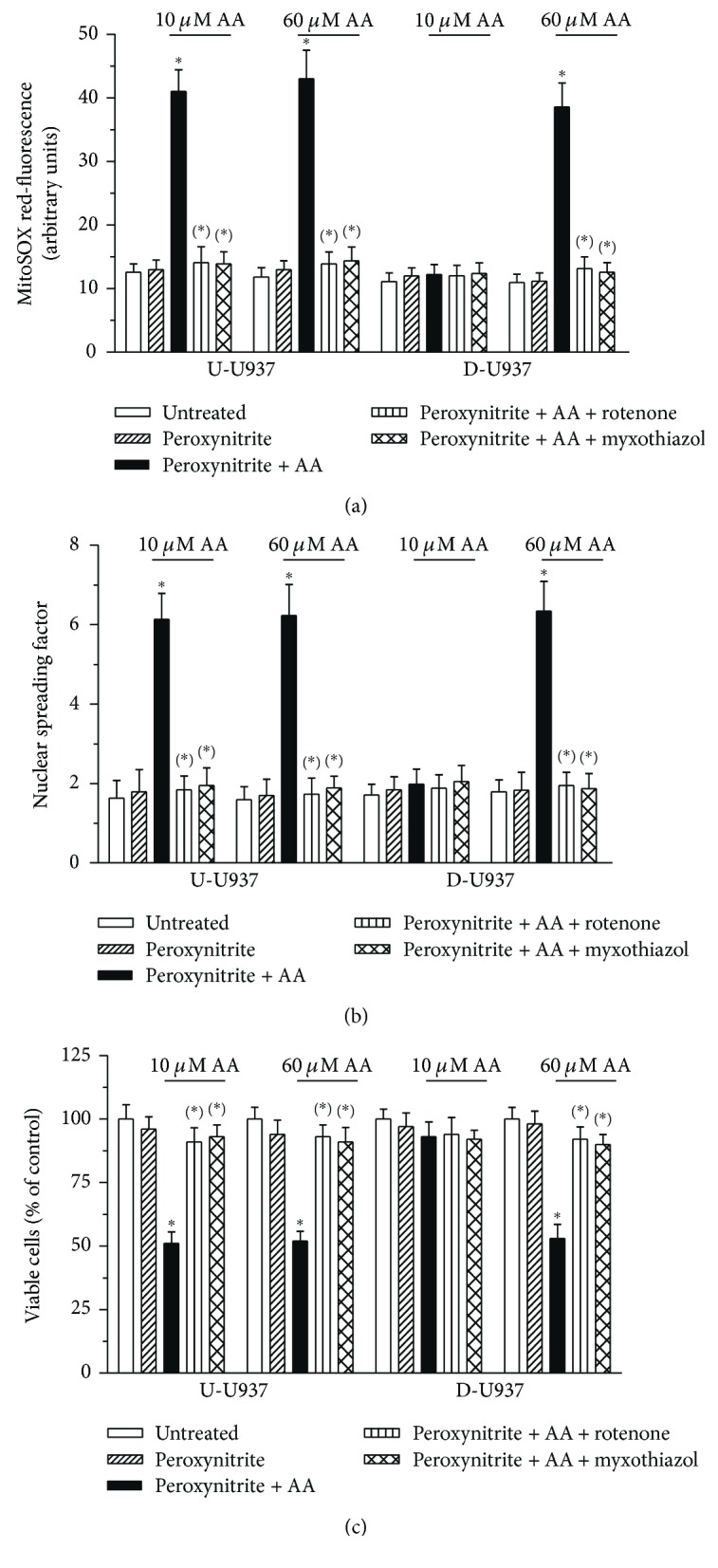
Mitochondrial superoxide formation, DNA strand scission, and cytotoxicity induced by peroxynitrite: different concentrations of AA are required to promote similar enhancing effects in undifferentiated and differentiated cells. Undifferentiated (U-U937) and differentiated (D-U937) cells preloaded with 10 *μ*M (U-U937) or 60 *μ*M (D-U937) AA in the presence of 100 *μ*M DTT were treated for 10 (a), 30 (b), or 60 min (c) with 40 *μ*M (U-U937) or 100 *μ*M (D-U937) peroxynitrite in the absence or presence of rotenone or myxothiazol. These inhibitors were given to the cells 3 min after peroxynitrite. After treatments, the cells were analyzed for MitoSOX red-fluorescence (a), DNA damage, (b) or cytotoxicity (c). Results represent the means ± SD calculated from at least 3 separate experiments. ^∗^*p* < 0.001 as compared to untreated cells, ^(∗)^*p* < 0.001 as compared to cells preexposed to AA and treated with peroxynitrite (one-way ANOVA followed by Dunnett's test).

On the bases of our previous studies, describing the different susceptibility of the two cell types to peroxynitrite [25], we adopted a protocol involving a preexposure to 10 or 60 *μ*M AA, followed by a treatment with 40 or 100 *μ*M peroxynitrite, of undifferentiated or differentiated cells, respectively. We found that peroxynitrite alone fails to produce effects in all of the above conditions. Evidence of rotenone or myxothiazol sensitive superoxide formation (Figure 5(a)), DNA strand scission (Figure 5(b)), and cytotoxicity (Figure 5(c)) was instead obtained in cells preexposed to AA and then treated with peroxynitrite for 10, 30, and 60 min, respectively. The concentrations of AA necessary to promote these enhancing effects were however different for the two cell types: 10 *μ*M AA indeed promoted maximal responses in the undifferentiated cells, with hardly any effect detected in their differentiated counterpart. In order to obtain similar enhancing effects, a preexposure of the differentiated cells to 60 *μ*M AA was necessary.

The results presented in this section provide evidence for specific functional implications of the reduced mitochondrial accumulation of AA observed in differentiated cells as a consequence of the reduced Vmax of SVCT2.

## 4. Discussion

In this study, we initially characterized the response of promonocytic U937 cells to a differentiating agent, DMSO, and provided evidence for the appearance of some characteristic features of circulating human monocytes [25, 34]. We then used these cells to address questions related to the impact of the differentiation process on the expression and functional activity of SVCT1 and SVCT2.

We found that U937 cell differentiation is associated with the downregulation of SVCT2 mRNA expression, in the absence of significant effects on SVCT1 mRNA. Notably, this event was detected in cells grown in the absence of vitamin C, thereby strongly suggesting that the observed inhibitory response is of specific biological relevance. Indeed, vitamin C deprivation, as discussed above, triggers opposite events associated with enhanced SVCT2 expression [2].

Additional relevant information is that the downregulated SVCT2 mRNA expression detected at day 4 of differentiation is not associated with the expected decrease in SVCT2 protein levels and cellular AA uptake. These findings, likely dependent on the half-life of the protein, nevertheless suggest that the differentiation process is accompanied by reduced SVCT2 expression, an event also expected to take place in circulating monocytes, since the plasma concentrations of vitamin C (about 60 *μ*M) are significantly higher than the Km of SVCT2. We can therefore formulate the hypothesis that circulating monocytes accumulate vitamin C through a mechanism regulated by an equilibrium defined by low levels of SVCT2 expression coupled with low levels of AA consumption.

In principle, DHA uptake through GLUTs might also contribute to the cellular accumulation of the vitamin [47], although it appears unlikely that this transport system can build up and maintain the high concentrations of vitamin C found in circulating monocytes [24]. The well-established notion that red blood cells only take up DHA but retain the same concentrations of the vitamin found in plasma [24, 47] indirectly emphasizes the relevance of SVCT2 in vitamin C transport in cells accumulating high concentrations of the vitamin, as monocytes.

Our results therefore indicate that the differentiation of promonocytes to monocytes is accompanied by reduced SVCT2 expression. A different scenario is instead to be expected upon monocyte recruitment in inflamed tissues, and with the ensuing differentiation of these cells to macrophages. Previous studies have indeed provided evidence for enhanced SVCT2 expression and activity, thereby implying a role for AA in the adaptive responses taking place during macrophage activation [20].

Our results on mitochondrial SVCT2 were somewhat different from those described above, as the differentiation process resulted in an apparent dichotomy between protein expression and transport activity. The differentiated cells were characterized by a remarkably enhanced mitochondrial SVCT2 immunoreactivity and a reduced mitochondrial accumulation of vitamin C, a notion also established in experiments in which AA transport was measured in isolated mitochondria. Kinetic studies revealed that reduced vitamin transport was dependent on a decreased Vmax of SVCT2 in the absence of significant changes in affinity, thereby implying that SVCT2 maintains the same low requirements for Na^+^ and Ca^2+^ described in our earlier studies performed in the undifferentiated U937 cells [6].

Our interpretation of these results is that the observed mitochondrial events represent an intermediate stage of the overall process of SVCT2 downregulation. More specifically, it appears that the inhibitory signal leading to reduced SVCT2 mRNA expression is followed by an intracellular redistribution of the SVCT2 protein, characterized by a significant accumulation in mitochondria. This latter event, however, had a negative impact on the mitochondrial SVCT2-transport activity. Although the mechanism leading to decreased Vmax of mitochondrial SVCT2 was not addressed in the present study, we consider possible and not mutually exclusive two different mechanisms. The first one is based on protein-protein interactions. Indeed, SVCT2 isoforms acting as dominant-negative inhibitors of high-affinity AA transporters have been previously identified [48]. The second mechanism is instead based on phosphorylation process, for example, driven by protein kinase C or protein kinase A, causing inhibition of high-affinity AA transport [49, 50]. Each of these explanations is compatible with the measured reduction in Vmax, in the absence of significant changes in Km of mitochondrial SVCT2.

For a correct interpretation of the results obtained in this study, we should once again remind that cultured cells overexpress high-affinity AA transporters to maximize their ability to take up the very low concentrations of the vitamin present in the culture media, which are not normally supplemented with vitamin C because of its poor stability. We previously observed [6] that sequential high-affinity transport through plasma and mitochondrial membrane SVCT2 in U937 cells exposed to low micromolar concentrations of vitamin C results in the accumulation of very high concentrations of AA in mitochondria. Given this premise, we should consider of likely physiological relevance the reduced Vmax of mitochondrial SVCT2 detected in the differentiated cells. The reduced need of mitochondrial vitamin C is probably linked to the intrinsic resistance of the differentiated cells to events associated with mitochondrial superoxide formation. This notion was clearly established in our previous studies indicating that differentiation of U937 cells to monocytes is associated with downregulation of the Ry receptor, a Ca^2+^ pool of critical importance for events leading to the mitochondrial uptake of the cation and to the ensuing formation of mitochondrial superoxide [34]. Importantly, human monocytes and macrophages do not express the Ry receptor [34], thereby suggesting that these cells, which contain millimolar levels of vitamin C, only need very low levels of mitochondrial SVCT2 expression.

The final part of this study was performed with the aim of identifying functional correlates of the different abilities of undifferentiated and differentiated cells to accumulate vitamin C in their mitochondria. For this purpose, we employed two different treatments associated with mitochondrial superoxide formation. The first one was based on a short-term exposure to a high concentration of BSO, a condition leading to about 40% decrease in GSH content and to a significant formation of reactive oxygen species, in both the undifferentiated and differentiated cells. The mitochondrial origin of these species was documented with the use of specific inhibitors. It was interesting to observe that the vitamin suppressed superoxide formation in both cell types, however at different concentrations. AA indeed produced maximal effects at 10 *μ*M in the undifferentiated cells and at 60 *μ*M in their differentiated counterpart.

We also employed a different strategy based on our previous findings indicating that preexposure of undifferentiated U937 cells to AA enhances their susceptibility to the deleterious effects mediated by peroxynitrite [11, 46]. These enhancing effects are based on the notion that mitochondrial AA increases the mitochondrial formation of superoxide mediated by peroxynitrite, via a Ca^2+^-independent mechanism associated with inhibition of complex III [30, 46]. As expected, preexposure to 10 *μ*M AA significantly increased mitochondrial superoxide formation and its downstream deleterious effects, induced by peroxynitrite in the undifferentiated cells. In order to obtain similar enhancing effects, the differentiated cells instead required preincubation with a greater concentration of AA.

Collectively, our results provide evidence for specific functional consequences of the reduced accumulation of AA in the mitochondria of the differentiated cells as a result of the reduced Vmax of mitochondria SVCT2.

In conclusion, while more studies are needed to understand the details of the effects under investigation, it nevertheless appears clear that the differentiation of promonocytic U937 cells to monocytes is accompanied by events resulting in downregulation of SVCT2 mRNA, which should then be followed by inhibition of SVCT2 protein expression. As an intermediate event detected at day 4 of differentiation, we observed an intracellular redistribution of the SVCT2 protein, under conditions in which the plasma membrane transport of the vitamin was still unaffected. More specifically, the SVCT2 protein was found to accumulate in mitochondria, in which decreased transport activity was unexpectedly detected as a consequence of reduced Vmax. In differentiated cells, mitochondrial uptake of AA was therefore significantly lower in comparison to the undifferentiated cells, as also demonstrated by measuring specific effects mediated by the mitochondrial fraction of the vitamin. Inhibition of the mitochondrial transport of AA through SVCT2 therefore anticipates the decline in SVCT2 protein expression elicited by the differentiation process, thereby suggesting a very limited contribution, if any, of an active mitochondrial transport of AA in circulating monocytes.
